# Atypical Clinical Presentation of Isolated Extraocular Muscle Sarcoid

**DOI:** 10.1155/2012/370258

**Published:** 2012-12-25

**Authors:** Wing Lung Alvin So, Thomas G. Hardy, Penelope McKelvie

**Affiliations:** ^1^Orbital, Plastic and Lacrimal Clinic, Royal Victorian Eye and Ear Hospital, 32 Gisborne Street, East Melbourne, VIC 3002, Australia; ^2^Department of Anatomical Pathology, St Vincent's Hospital, 41 Victoria Parade, Fitzroy, VIC 3065, Australia

## Abstract

A case of painless isolated extraocular muscle sarcoid and a literature review are presented. The atypical features in our case include a lack of overt inflammatory symptoms and signs, such as pain, ptosis, and diplopia. The presentation of minimal symptoms without improvement warrant a biopsy to establish the diagnosis and to administer appropriate treatment in order to prevent potential long-term complications from sarcoidosis.

## 1. Introduction

Sarcoidosis is an idiopathic multisystem disorder, most commonly affecting the lung, skin, lymph nodes, and the eye. It is more common in Caucasians of Northern European descent and African-Americans, aged between 20 and 40 years [1]. Histologically, the characteristic feature is noncaseating granuloma.

Eye involvement is common in the anterior segment, and about 80% of patients develop uveitis. Lacrimal gland and orbital fat may be affected. Isolated extraocular muscle involvement is rare, and most patients in the literature had mass effect and inflammatory symptoms including pain and diplopia [2, 3]. We present a case of isolated extraocular muscle involvement by sarcoid with neither diplopia nor significant inflammatory symptoms. 

## 2. Case Report

A 36-year-old Caucasian female presented with two-month history of a vague lump below her left eye. She had no pain or diplopia. She had no past history of sarcoidosis, malignancy, injury, or autoimmune disorders including Graves' disease. She did not take any medication previously and stopped smoking four weeks before presentation. Initially, a local infection was suspected, and treatment with systemic and local antibiotics was commenced prior to presentation. However, there was no symptomatic improvement, and she was referred to an orbital surgeon (TGH). 

On examination, a nontender lump with mild swelling was noted in the inferonasal quadrant of her left orbit. Visual acuity was normal. There was no proptosis or nonaxial globe displacement. Full range of ocular movements was achieved with no diplopia or discomfort. Her ocular and fundus examinations were normal. Cardiorespiratory examination was unremarkable. No skin rashes nor enlarged lymph nodes were noted.

**Figure 1:**
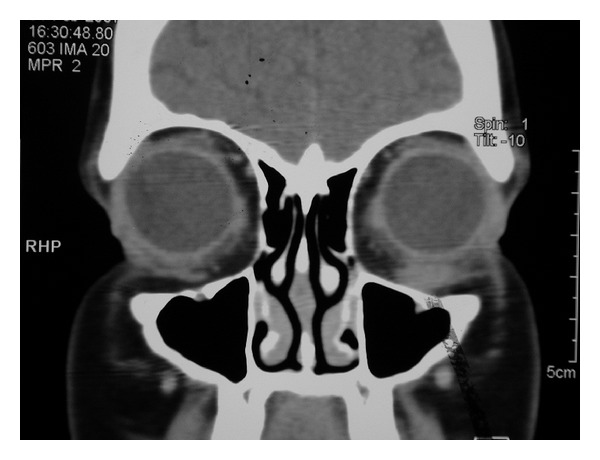
Coronal computerised tomography scan showing diffuse swelling of the left inferior oblique muscle.

**Figure 2:**
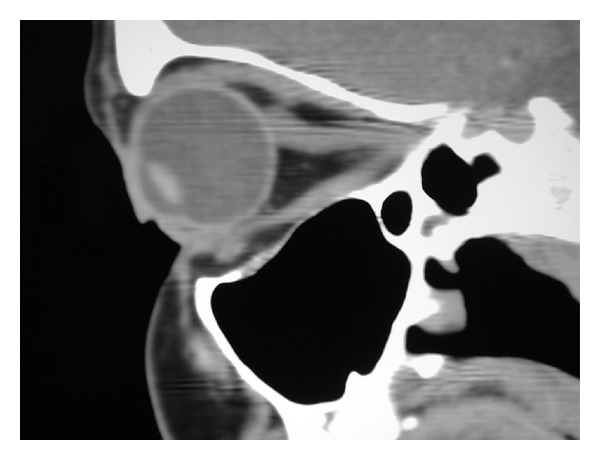
Parasagittal view showing normal right orbital contents.

**Figure 3:**
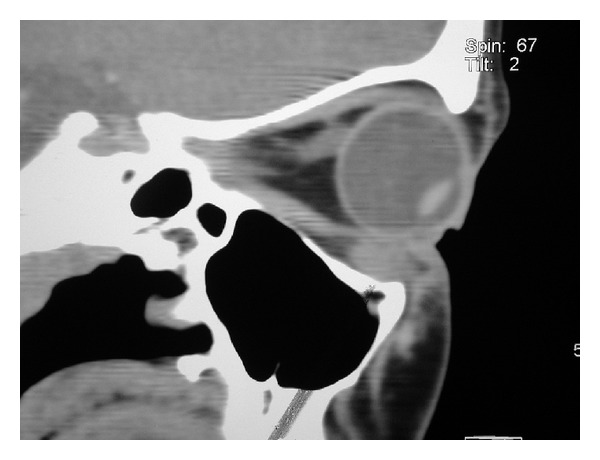
Parasagittal view showing swelling in left inferior oblique.

**Figure 4:**
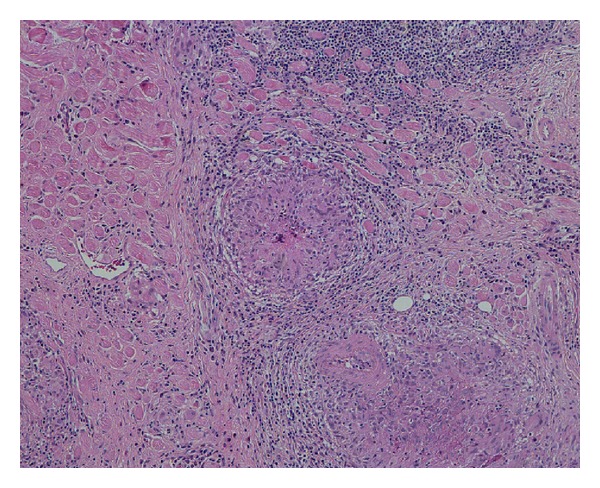
Histological staining with haematoxylin and eosin (10x magnification) showing multiple focally necrotising sarcoid-type granulomata.

**Figure 5:**
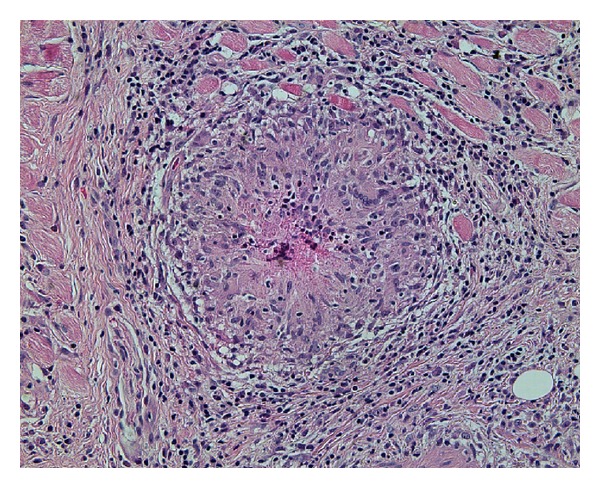
Higher magnification of 20x view showing multinucleated giant cells within the muscle and focal endomysial infiltrates of lymphocytes and plasma cells, consistent with a diagnosis of *orbital sarcoid*, although CT chest and ongoing followup have excluded systemic sarcoidosis.

Computerised tomography (CT) revealed diffuse swelling of her left inferior oblique muscle with no other abnormalities (Figures 1, 2, and 3). Full blood examination, liver function test, thyroid function test, and the levels of erythrocyte sedimentation rate, C-reactive protein, serum electrolytes, creatinine, creatinine kinase, angiotensin converting enzyme (ACE), and anti-neutrophil cytoplasmic antibodies (ANCAs) were normal. Given her atypical presentation, a biopsy of the left inferior oblique was performed. Histology revealed multiple focally necrotising sarcoidal granulomata with multinucleated giant cells within the muscle and focal endomysial infiltrates of lymphocytes and plasma cells (Figures 4 and 5). No vasculitis was noted, and special stains for fungi and acid-fast bacilli were negative. The histopathological diagnosis was that of sarcoidal granulomatous myositis with focal necrosis of granulomata. The differential diagnoses included sarcoidosis, tuberculosis, drugs, other infections, granulomatous polymyositis, paraneoplastic, and idiopathic orbital myositis. Her symptoms resolved following a short course of low dose prednisolone, apart from the subtle short-term left inferior oblique underaction as a result of the biopsy. Referrals were made to an infectious disease physician and a respiratory physician to assess for underlying aetiology. Extrapulmonary tuberculosis (TB) was excluded, as the patient had had no known exposure to TB, and her chest X-Ray and Quantiferon Gold test were normal. Although histopathology of the extraocular muscle showed multiple sarcoidal granulomata, there was no evidence of systemic or pulmonary involvement of sarcoidosis. High resolution chest CT showed only slight peribronchial thickening in basal segments and borderline bronchiectasis inone or two airways. Follow-up orbital CT showed complete resolution of the left inferior oblique swelling, which was symmetrical with the right side muscle. Ongoing followup over three years has shown no evidence of recurrence or systemic manifestation of sarcoidosis.

## 3. Discussion

Sarcoidosis is an inflammatory disorder which most commonly affects the pulmonary system. Dry cough and dyspnoea are common symptoms, as are constitutional symptoms such as weight loss and fatigue. Lymphadenopathy may occur. Ocular manifestations are not uncommon, particularly uveitis and retinitis. Isolated extraocular muscle involvement with minimal clinical symptoms and signs is extremely rare, and there have only been several reports of extraocular muscle sarcoid previously, most of which reported overt symptoms of inflammation such as pain, as well as abnormalities in ocular examination [4–10]. In addition, systemic involvement of sarcoidosis has been found in most of these cases. To our knowledge, this is the first case of isolated extraocular muscle sarcoid with no diplopia, pain, nor other symptoms of inflammation.

The diagnosis of orbital sarcoid can be made in tissue biopsy. Chest CT should be performed to exclude pulmonary involvement. ACE level is helpful, although a normal result does not exclude sarcoid [4]. Many cases of sarcoidosis resolve with no long-term complications, however, some patients may develop respiratory and/or renal failure. Accurate assessment and treatment is helpful to halter this process.

Clinically, orbital sarcoid may be confused with Graves' orbitopathy, particularly in this case given the subacute and painless nature. However, it can be excluded by the absence of any other features of Graves' orbitopathy, such as lid retraction, and normal thyroid function. Furthermore, isolated inferior oblique involvement by Graves orbitopathy is rare [11].

Other possible causes include Wegener's granulomatosis and infections such as Coxsackie virus B2 and tuberculosis [12]. Extraocular involvement has been reported as a first presentation of Wegener's granulomatosis, but this can be excluded by a normal c-ANCA level and lack of vascular inflammation in tissue biopsy [13]. In addition, our patient had no sinus symptoms, which commonly precede orbital involvement in Wegener's granulomatosis. Orbital myositis associated with other systemic inflammatory diseases, for example, systemic lupus erythematosus and Crohn's disease, usually have a past history of the corresponding disease [12, 14]. Currently, extrapulmonary tuberculosis has an increasing incidence in developed countries [15]. It should be considered in this case, as there were no acute inflammatory features clinically, and necrotising granulomata were present in histopathological examination. However, the patient had no risk factors for tuberculosis, and chest X-ray and Quantiferon Gold test were normal [16]. Ziehl-Neelsen and PAS stains were also negative for fungi and acid-fast bacilli in biopsy. 

 Extraocular involvement in lymphoid tumours and metastasis has been reported, although lymphoid markers were negative in our case. In addition, the histopathological findings were not consistent with a lymphoproliferative disorder [17]. Paraneoplastic syndrome and Tolosa-Hunt syndrome usually have variable cranial nerve involvement [18, 19]. Finally, idiopathic granulomatous myositis may have similar clinical features and histopathology, although there is ongoing debate whether it is a separate entity or merely a type of myo-restricted sarcoidosis [20]. 

The treatment of our patient included only an 8-day course of dose-reducing oral prednisolone, unlike previous case reports in which surgical debulking and/or radiotherapy were often required to reduce mass effect [10]. Our patient had had no further symptoms despite more than 3 years of followup.

In conclusion, we have reported an atypical presentation of isolated extraocular muscle sarcoid, in a patient who had no overt inflammatory symptoms. Early biopsy of orbital masses not responding to initial treatment is important to enable accurate diagnosis and appropriate management, and to reduce the likelihood of long-term complications from sarcoid.
